# Letter to the Editor on “Development and validation of PEYRO-Q: a novel multidimensional patient-reported outcome measure for Peyronie’s disease”

**DOI:** 10.1093/sexmed/qfag078

**Published:** 2026-10-21

**Authors:** Adil Emrah Sonbahar, Mehmet Gokhan Culha

**Affiliations:** Department of Urology, Izmir Katip Celebi University, Atatürk Training & Research Hospital, Izmir 35360, Türkiye; Department of Urology, University of Health Sciences, Prof. Dr. Cemil Tascioglu City Hospital, Istanbul 34384, Türkiye

We read with interest the article by Cocci et al. on the development of the PEYROnie's disease Questionnaire (PEYRO-Q), a novel patient-reported outcome measure (PROM) for Peyronie’s disease.^1^ While a more inclusive tool is needed, we have serious concerns about the methodological quality of this validation study. Despite citing COnsensus-based Standards for the selection of health Measurement INstruments (COSMIN) guidelines, several fundamental psychometric properties remain unaddressed, making the PEYROQ unsuitable for clinical or research use.

First, the study lacks proper cross-cultural validation. Although described as multicenter and international, 84.5% of participants were Italian, and the questionnaire was developed in Italian. No details are provided on translation, back-translation, or cultural adaptation into other languages (eg, French, German, Spanish). According to international standards, a PROM cannot be used across linguistic populations without formal linguistic validation.^2^ The current dataset essentially validates only an Italian version.

Second, the factor structure is inadequately examined. The authors propose a 10-domain structure but performed an exploratory factor analysis that extracted only 3 factors. Crucially, they do not report item loadings or which items loaded onto which factors. Moreover, a confirmatory factor analysis (CFA)—mandatory for testing a prespecified structure—was entirely omitted.^3^ The mismatch between the empirical factor structure and the theoretical model suggests poor construct validity.

Third, concurrent validity testing is absent. The authors state they could not use the Peyronie's Disease Questionnaire (PDQ) due to permission issues, but this does not justify omitting comparisons with widely available, validated instruments such as the International Index of Erectile Function (IIEF) or Hospital anxiety and depression scale (HADS). A new PROM must demonstrate its relationship to existing gold standards to be considered valid.

Fourth, the reliability analysis contains a fundamental statistical error. The authors report test–retest reliability using Cronbach’s alpha. Cronbach’s alpha measures internal consistency (homogeneity of items), not temporal stability. Test–retest reliability must be assessed using the intraclass correlation coefficient (ICC) or weighted kappa.^4^ Reporting Cronbach’s alpha for repeated administrations is conceptually incorrect and invalidates the reliability claims.

Finally, the PEYROQ lacks responsiveness and Minimal clinically important differences (MCID) data. A PROM intended for longitudinal monitoring must demonstrate sensitivity to clinically meaningful change over time and establish the minimal clinically important difference. Without these properties, the instrument cannot be used in treatment trials or for individual patient monitoring.

In conclusion, this study does not meet minimum psychometric standards for PROM validation. We strongly recommend that the authors conduct a proper validation study including CFA, cross-cultural adaptation, convergent validity testing against established instruments, correct test–retest reliability using ICC, and longitudinal responsiveness data before claiming the PEYROQ is ready for use.

Accordingly, until these essential steps are completed and published, the PEYROQ should not be used in clinical research or treatment trials. Applying an unverified instrument risks generating misleading data and undermining study integrity and cross-study comparability. We urge that adoption of the PEYROQ be deferred until a methodologically rigorous revalidation is demonstrated.
